# Real-world evidence from a University Hospital system regarding the uptake of adjuvant pertuzumab and/or neratinib before and after their FDA approval

**DOI:** 10.1007/s10549-021-06132-8

**Published:** 2021-02-24

**Authors:** Ericson Stoen, Jodi Kagihara, Elena Shagisultanova, Christine M. Fisher, Andrew Nicklawsky, Peter Kabos, Virginia F. Borges, Jennifer R. Diamond

**Affiliations:** 1 Department of Internal Medicine, University of Colorado School of Medicine, 12401 East 17th Avenue, Mailstop F-782, Aurora, CO 80045 USA; 2 Division of Medical Oncology, University of Colorado School of Medicine, Aurora, CO USA; 3 Department of Radiation Oncology, University of Colorado School of Medicine, Aurora, CO USA; 4 Department of Pediatrics, University of Colorado School of Medicine, Aurora, CO USA

**Keywords:** Breast cancer, HER2, Pertuzumab, Neratinib, Adjuvant therapy

## Abstract

**Purpose:**

Adjuvant pertuzumab and neratinib are independently FDA-approved for treatment of early-stage HER2-positive breast cancer in combination with or following trastuzumab for one year, respectively. Both agents reduce the risk of recurrence; however, the absolute benefit is modest for many patients with added risk of adverse effects. The purpose of this study was to evaluate the clinical use of adjuvant pertuzumab and neratinib in patients with early-stage HER2-positive breast cancer.

**Methods:**

Patients diagnosed with stage I–III HER2-positive breast cancer treated with trastuzumab at four University of Colorado Health hospitals between July 2016 and April 2019 were identified. Patient demographics, cancer stage, treatment, and administration of pertuzumab and/or neratinib were obtained.

**Results:**

We identified a total of 350 patients who received adjuvant trastuzumab for stage I–III HER2-positive breast cancer; 253 (73.1%) had tumors that were ≥ T2 or node-positive disease. The rate of adjuvant pertuzumab use increased following FDA approval; pertuzumab was administered to the majority of patients with node-positive HER2-positive breast cancer. The use of adjuvant pertuzumab was associated with younger age, premenopausal status, and node-positive disease. Rates of administration of adjuvant neratinib were lower, with only 15.2% of patients receiving this therapy within 3 months of completing adjuvant trastuzumab.

**Conclusion:**

In our cohort of patients treated within a diverse healthcare network, the majority of patients with node-positive HER2-positive breast cancer received adjuvant pertuzumab following FDA approval. The use of adjuvant neratinib was less common, potentially as a result of adverse effects, prolongation of therapy, previous administration of adjuvant pertuzumab, and modest benefit.

## Introduction

Breast cancer is the most common cancer in women and approximately 20% of breast cancers overexpress human epidermal growth factor receptor 2 (HER2) [1–3]. HER2-positive breast cancer was historically associated with a worse prognosis; however, the addition of trastuzumab to adjuvant chemotherapy significantly reduced the risk of disease recurrence and death [4–8]. Both pertuzumab and neratinib have been independently shown to further reduce the risk of recurrence in patients with HER2-positive breast cancer treated with chemotherapy and trastuzumab for one year, though the magnitude of benefit is much smaller than the initial benefit gained from targeting HER2 with the addition of trastuzumab [9, 10].

Pertuzumab is a humanized recombinant anti-HER2 monoclonal antibody that inhibits dimerization of HER2 with other HER family receptors and is synergistic with trastuzumab [11–13]. The addition of pertuzumab to docetaxel and trastuzumab in the first-line, HER2-positive metastatic setting led to an improvement in progression-free survival (PFS) and overall survival (OS) and remains the standard of care for these patients [12]. In the neoadjuvant setting, the addition of pertuzumab to trastuzumab-containing chemotherapy led to improvements in pathologic complete response (pCR) and disease-free survival (DFS) and remains the standard of care for neoadjuvant therapy in high-risk patients [13, 14]. In patients with node-positive or high-risk node-negative operable HER2-positive breast cancer, the APHINITY trial found that the addition of pertuzumab improved the 3-year rate of invasive disease-free survival (iDFS) from 93.2% in the placebo group to 94.1% in the pertuzumab group (HR 0.81; 95% CI 0.66–1.00, *P* = 0.045). The benefit was greater in patients with node-positive disease, with a 3-year iDFS rate of 90.2% in the placebo group and 92.0% in those receiving pertuzumab (HR 0.77; 95% CI 0.62–0.96, *P* = 0.02). Cardiac events were low in both groups and diarrhea was more common with pertuzumab [9]. With 6-year follow-up, the addition of pertuzumab did not result in a statistically significant difference in OS, but continued follow-up is planned [15].

Neratinib is an oral small-molecule tyrosine kinase inhibitor of HER1, HER2, and HER4 with single-agent and combination activity in patients with metastatic HER2-positive breast cancer previously treated with trastuzumab [10, 16–18]. In the neoadjuvant setting, the addition of neratinib to standard chemotherapy with trastuzumab resulted in an improvement in the pCR rate in HER2-positive, hormone receptor-negative patients in the I-SPY2 trial [18]. Based on these results, neratinib was evaluated in the adjuvant setting in the phase III, randomized, placebo-controlled ExteNET study, which assessed the benefit of addition of 1 year of adjuvant neratinib after standard chemotherapy and trastuzumab in patients with early-stage, high-risk HER2-positive breast cancer. The addition of neratinib resulted in an improvement in the 5-year iDFS rate versus placebo (90.2% vs. 87.7%, HR 0.73; 95% CI 0.57–0.92, *P* = 0.0083). The magnitude of benefit in 5-year iDFS was greater in the subgroup of patients with hormone receptor-positive disease (HR 0.60; 95% CI 0.43–0.83) compared to a nonsignificant difference in patients with hormone receptor-negative disease (HR 0.95; 95% CI 0.66–1.35) [10]. In the ExteNET trial, patients treated with adjuvant neratinib had a significantly higher rate of grade 3 diarrhea compared to placebo (40% versus 2%). No patients enrolled in ExteNET received prior pertuzumab and no patients enrolled in APHINITY received adjuvant neratinib [9, 10].

Given the simultaneous approval of both pertuzumab and neratinib for the adjuvant treatment of high-risk, early-stage, HER2-positive breast cancer, the added toxicity with these agents, and the modest improvement in recurrence rates overall, the decision to incorporate these agents into standard practice is complex. The purpose of this study was to evaluate the use of adjuvant pertuzumab and neratinib in patients with high-risk, early-stage HER2-positive breast cancer within the University of Colorado Health system.

## Methods

Female patients aged 18–84 years old diagnosed with stage I–III HER2-positive breast cancer treated with trastuzumab at multiple centers within the University of Colorado Health system between July 2016 and April 2019 were identified using an electronic data capture system. This study was performed following local institutional review board approval and all data were stored in a secure online database. HER2-positivity was defined by local pathology report using ASCO/CAP guidelines [19]. Patients pregnant at the time of treatment were excluded. Patients must have had at least two visits with a provider within the system to ensure adequate follow-up. Patients received treatment at four centers in the University of Colorado Health system. Chart review was performed for all patients using the electronic medical record. Baseline characteristics including age; date of diagnosis; stage at diagnosis (highest clinical or pathologic stage); estrogen receptor expression (as assessed by local pathology report); menopausal status (by physician documentation); cancer treatment including surgery, radiation, chemotherapy; and receipt of adjuvant pertuzumab and/or neratinib were collected. Patients receiving at least one dose of pertuzumab following the completion of neoadjuvant or adjuvant chemotherapy were included as having received adjuvant pertuzumab. The duration of adjuvant pertuzumab was determined and receipt of 12 months was considered a complete course (including neoadjuvant or adjuvant pertuzumab administered in combination with chemotherapy). Patients receiving any duration of neratinib within 3 months of completing adjuvant trastuzumab were considered as having received adjuvant neratinib. Given the high incidence of neratinib-induced diarrhea, receipt of concomitant anti-diarrheal prophylaxis was also collected.

Patient characteristics were analyzed using percentages for categorical variables and mean for continuous variables. Associations between patient characteristics and administration of pertuzumab or neratinib were performed using the 2-sided Chi-squared test for categorical variables. For variables with small sample size, data were consolidated and reported as pooled. An alpha value of 0.05 was used for all statistical analysis, which was considered statistically significant.

## Results

### Baseline characteristics

In our cohort, 350 patients were identified with stage I–III HER2-positive breast cancer who received trastuzumab and were included in our analysis. Of those, 346 had adequate follow-up to be included in our dataset. In order to focus our analysis on patients with high-risk, HER2-positive breast cancer, our analysis was limited to the 253 patients (73.1%) with tumor stage ≥ T2 or node-positive disease at diagnosis. Patients with disease that was T1 or node-negative were not included in our analysis.

Tables 1 and 2 depict baseline patient characteristics. The mean age of patients with high-risk, early-stage HER2-positive breast cancer was 53.2 years (range 24–82), and 171 of 253 (67.6%) had node-positive disease. The majority of patients had hormone receptor-positive disease (172/253, 68.0%), and 133 of 253 (52.6%) were postmenopausal. Neoadjuvant chemotherapy was administered to 73.9% of patients in this cohort (187/253 patients).

### Patients receiving adjuvant pertuzumab

High-risk, early-stage HER2-positive breast cancer patients receiving adjuvant pertuzumab were younger than patients who did not receive adjuvant pertuzumab (mean age of 48.4 compared to 55.8 years, *P* < 0.0001) (Table 1). There was also a higher incidence of utilization of adjuvant pertuzumab in premenopausal patients (*P* = 0.0053). Hormone receptor positivity was not associated with administration of adjuvant pertuzumab (70.7% [65/92] versus 66.5% [107/161], *P* = 0.4917), nor was the likelihood of pCR after neoadjuvant chemotherapy, if given (42.5% [31/73] versus 44.7% [51/114], *P* = 0.7601). Among patients that received adjuvant pertuzumab, 76.1% (70/92) had node-positive disease. Of all high-risk patients studied, 36.4% (92/253) received adjuvant pertuzumab, including 39.8% (68/171) of patients with node-positive disease and 26.8% (22/82) of patients with node-negative, ≥ T2 disease (Table 2).

**Table 1:** Baseline characteristics of patients with high-risk early-stage HER2-positive breast cancer receiving or not receiving adjuvant pertuzumab

	All patients (*N* = 253)	Received pertuzumab (*N* = 92)	Did not receive pertuzumab (*N* = 161)	*P*-value*
**Age (years)**				< 0.0001
Mean	53.2	48.4	55.8	
Range Standard deviation	24–82 13.31	31–73 10.99	24–82 13.78	
**Practice Setting**				0.0442
Academic	94 (37.2%)	42 (45.7%)	53 (32.9%)	
Community	159 (62.8%)	50 (54.3%)	108 (67.1%)	
**AJCC Tumor Size**				0.1857
T1	43 (17.0%)	13 (14.1%)	31 (19.2%)	
T2	158 (62.5%)	53 (57.6%)	105 (65.2%)	
T3	34 (13.4%)	17 (18.5%)	17 (10.6%)	
T4	16 (6.3%)	8 (8.7%)	7 (4.3%)	
TX	2 (0.8%)	1 (1.1%)	1 (0.6%)	
**Nodal Status**				0.0356
N0	82 (32.4%)	22 (23.9%)	62 (38.5%)	
N1	139 (54.9%)	52 (56.5%)	85 (52.8%)	
N2	15 (5.9%)	9 (9.8%)	6 (3.7%)	
N3	17 (6.7%)	9 (9.8%)	8 (5.0%)	
**Hormone Status**				0.4917
ER and/or PR-positive	172 (68.0%)	65 (70.7%)	107 (66.5%)	
ER/PR-negative	81 (32.0%)	27 (29.3%)	54 (33.5%)	
**Menopausal Status**				0.0053
Premenopausal	97 (38.3%)	47 (51.1%)	51 (31.7%)	
Postmenopausal	133 (52.6%)	36 (39.1%)	96 (59.6%)	
Unknown	23 (9.1%)	9 (9.8%)	14 (8.7%)	
**Received Radiation**				0.1134
Yes	193 (76.3%)	75 (81.5%)	117 (72.7%)	
No	60 (23.7%)	17 (18.5%)	44 (27.3%)	
**Received Neoadjuvant Chemotherapy**				
Yes	187 (73.9%)	73 (79.3%)	114 (70.8%)	
No	66 (26.1%)	19 (20.7%)	47 (29.2%)	
**pCR After Neoadjuvant Chemotherapy**				0.7601
Yes	83 (44.4%)	31 (42.5%)	51 (44.7%)	
No	104 (55.6%)	42 (57.5%)	63 (55.3%)	

*P-value calculated using Chi-squared analysis comparing pertuzumab to no pertuzumab groups

**Table 2:** Use of adjuvant pertuzumab by cancer stage

	Node-positive and/or tumor ≥ T2 (*N* = 253)	Node-positive (*N* = 171)	Node-negative, tumor ≥ T2 (*N* = 82)
Adjuvant pertuzumab	92 (36.4%)	70 (40.9%)	22 (26.8%)
No adjuvant pertuzumab	161 (63.6%)	101 (59.1%%)	60 (73.2%)

**Fig. 1:**
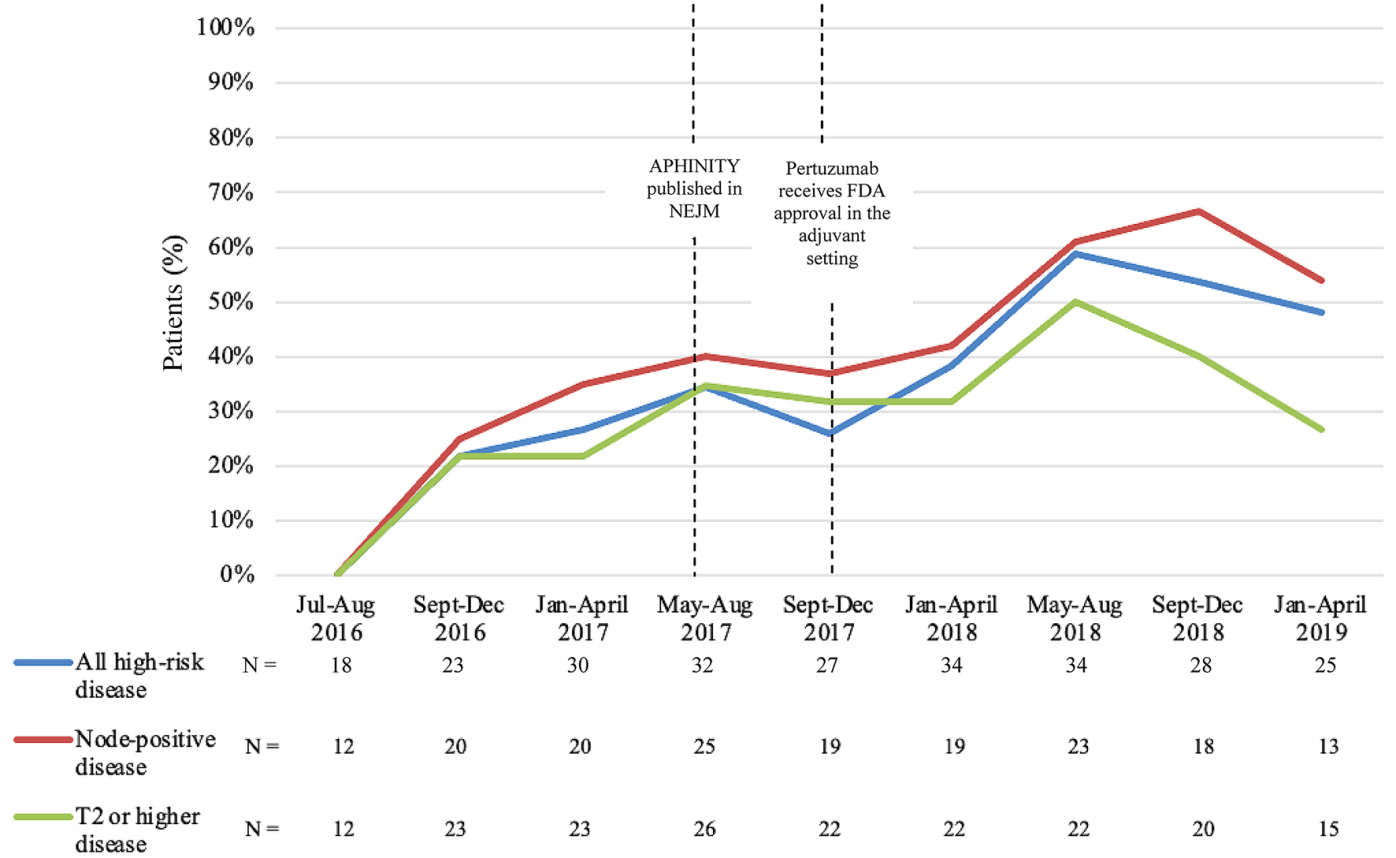
Rates of initiation of adjuvant pertuzumab in patients with high-risk, early-stage HER2-positive breast cancer over time

The rate of adjuvant pertuzumab administration increased over time from July 2016 to December 2018 following the publication of the APHINITY trial results in July 2017 and the FDA approval of adjuvant pertuzumab in high-risk early-stage breast cancer in December 2017 (Fig. 1). At the peak of administration in our cohort, approximately two-thirds of patients with node-positive, HER2-positive disease received adjuvant pertuzumab. The average duration of therapy was 10.7 months and 83.7% of patients received at least 8 months of therapy.

### Patients receiving adjuvant neratinib

The mean age of patients with high-risk, early-stage HER2-positive breast cancer who received adjuvant neratinib was 49.1 compared to 53.8 years for those who did not (*P* = 0.0679) (Table 3). Hormone receptor status did not correlate with neratinib use; however, there was a trend toward increased use in hormone receptor-positive patients. Patients receiving adjuvant neratinib were less likely to have achieved pCR following neoadjuvant chemotherapy (24.0% [6/25] versus 47.2% [77/163], *P* = 0.0297).

**Table 3:** Baseline characteristics of patients with high-risk early-stage HER2-positive breast cancer receiving or not receiving adjuvant neratinib

	All patients (*N* = 253)	Received neratinib (*N* = 30)	Did not receive neratinib (*N* = 223)	*P*-value*
**Age (years)**				0.0679
Mean	53.2	49.1	53.8	
Range	24–82	29–77	24–82	
Standard deviation	13.31	13.13	13.26	
**Location of Care**				0.0871
Academic	94 (37.2%)	17 (56.7%)	77 (34.5%)	
Community	159 (62.8%)	13 (43.3%)	146 (65.5%)	
**AJCC Tumor Size**				0.0027
T1	43 (17.0%)	4 (13.3%)	39 (17.5%)	
T2	158 (62.5%)	13 (43.3%)	145 (65.0%)	
T3	34 (13.4%)	11 (36.7%)	23 (10.3%)	
T4	16 (6.3%)	2 (6.7%)	14 (6.3%)	
TX	2 (0.8%)	0	2 (0.9%)	
**Nodal Status**				0.0180
N0	82 (32.4%)	4 (13.3%)	78 (35.0%)	
N1	139 (54.9%)	20 (66.7%)	119 (53.4%)	
N2	15 (5.9%)	1 (3.3%)	14 (6.3%)	
N3	17 (6.7%)	5 (16.7%)	12 (5.4%)	
**Hormone Status**				0.2776
ER and/or PR-positive	172 (68.0%)	23 (76.7%)	149 (66.8%)	
ER/PR-negative	81 (32.0%)	7 (23.3%)	74 (33.2%)	
**Menopausal Status**				0.1504
Premenopausal	97 (38.3%)	16 (53.3%)	81 (36.3%)	
Postmenopausal	133 (52.6%)	13 (43.3%)	120 (53.8%)	
Unknown	23 (9.1%)	1 (3.3%)	22 (9.9%)	
**Received Radiation**				0.0599
Yes	193 (76.3%)	27 (90.0%)	166 (74.4%)	
No	60 (23.7%)	3 (10.0%)	57 (25.6%)	
**Received Neoadjuvant Chemotherapy**				
Yes	187 (73.9%)	25 (83.3%)	162 (72.6%)	
No	66 (26.1%)	5 (16.7%)	61 (27.4%)	
**pCR After Neoadjuvant Chemotherapy**				0.0297
Yes	83 (44.4%)	6 (24.0%)	77 (47.5%)	
No	104 (55.6%)	19 (76.0%)	85 (52.5%)	

*P-value calculated using Chi-squared analysis comparing neratinib to no neratinib groups

**Fig. 2:**
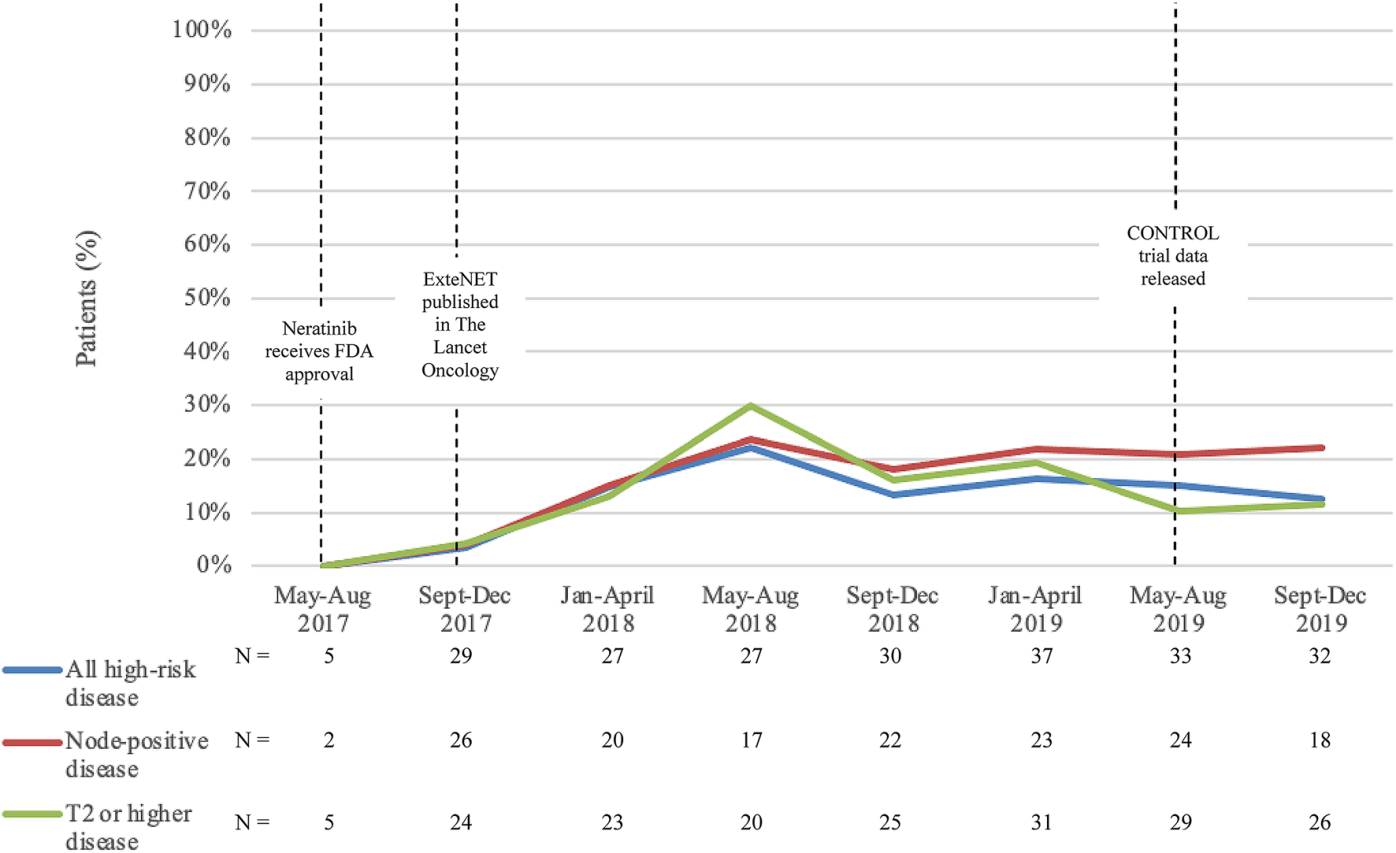
Rates of initiation of adjuvant neratinib in patients with high-risk, early-stage HER2-positive breast cancer over time

In our dataset of patients with ≥ T2 or node-positive disease, only 30 of 253 patients (11.9%) received adjuvant neratinib. Among patients with node-positive disease, 15.2% (26/171) received adjuvant neratinib; among patients with ≥ T2, node-negative disease, 4.9% (4/82) received adjuvant neratinib (Table 4). The majority of patients (86.7%) receiving adjuvant neratinib had node-positive disease. Rates of administration of adjuvant neratinib increased steadily following the publication of the ExteNET trial results in November 2017, reaching a peak in May 2018 when approximately 30% of patients with high-risk, early-stage HER2-positive breast cancer were treated with adjuvant neratinib (Fig. 2). No patients received adjuvant neratinib prior to FDA approval. For patients receiving adjuvant neratinib, the average time from completion of adjuvant trastuzumab to initiation of adjuvant neratinib was 2.97 months. All patients receiving adjuvant neratinib were also prescribed diarrheal prophylaxis with loperamide. Additional anti-diarrheal agents included colestipol (66.7%), atropine/diphenoxylate (Lomotil) (3%), cholestyramine (6%), and budesonide (3%).

**Table 4:** Use of adjuvant neratinib by cancer stage

	Node-positive and/or tumor ≥ T2 (*N* = 253)	Node-positive (*N* = 171)	Node-negative, tumor ≥ T2 (*N* = 82)
Adjuvant neratinib	30 (11.9%)	26 (15.2%)	4 (4.9%)
No adjuvant neratinib	223 (88.1%)	145 (84.8%)	78 (95.1%)

We identified 14 patients (5.5%) of the 253 identified with high-risk, early-stage HER2-positive breast disease who received both adjuvant pertuzumab and adjuvant neratinib. These patients had many high-risk features including younger age (average 42.9 years); T2 or higher tumors (14/14), and node-positivity (12/14, 85.7%). The majority of patients receiving both pertuzumab and neratinib were HR-positive (12/14, 85.7%) and of the 11 that received neoadjuvant chemotherapy, 7 (63.6%) did not achieve a pCR.

## Discussion

In our study, we evaluated the practice patterns for the use of adjuvant pertuzumab and neratinib in patients with high-risk, early-stage HER2-positive breast cancer as defined as ≥ T2 or node-positive disease. We found that the majority of patients with node-positive disease went on to receive adjuvant pertuzumab following FDA approval in the adjuvant setting, and patients with node-negative, ≥ T2 disease were less likely to receive this treatment. Patients receiving adjuvant pertuzumab were younger, and more likely to be premenopausal and node-positive. Adjuvant neratinib was less commonly used in this cohort and was administered to 11.9% of patients overall and 15.2% of patients with node-positive disease. There was a trend toward higher use in patients with HR-positive disease; however, the number of patients receiving adjuvant neratinib was small in our dataset, which limited this analysis.

We observed that patients with node-positive disease were most likely to receive adjuvant pertuzumab, consistent with the benefit observed in this patient population in the APHINITY trial [9]. The observed practice patterns are consistent with both NCCN and ASCO guidelines recommending the consideration of adjuvant pertuzumab in patients with node-positive, HER2-positive breast cancer [20, 21].

Interestingly, we observed a relatively high rate of administration of adjuvant pertuzumab in high-risk patients prior to its FDA approval in the adjuvant setting. One reason for this may be the magnitude of benefit observed with the addition of pertuzumab to treatment regimens in the metastatic and neoadjuvant settings which supported the evaluation of pertuzumab in the APHINITY trial. As pertuzumab was commercially available at the time and used in the neoadjuvant setting for many of these patients, providers likely had the opportunity to continue pertuzumab in the adjuvant setting for patients identified as high risk of recurrence while awaiting the results of APHINITY. Following publication of the APHINITY trial results, rates of administration of adjuvant pertuzumab further increased in node-positive patients. In the final quarter of our analysis, a decline in the rate of use of adjuvant pertuzumab was observed which correlated with publication of the KATHERINE trial and a change in our standard of care to administer ado-trastuzumab emtansine in the adjuvant setting for patients who received neoadjuvant chemotherapy without pCR [22].

The mean duration of adjuvant pertuzumab for patients receiving it in our study was 10.7 months and the vast majority of patients received at least 8 months of therapy. Treatment discontinuation prior to the planned 12 months of therapy most commonly occurred due to treatment-related toxicities. The most frequently reported high-grade adverse events in clinical trials of pertuzumab in combination with chemotherapy and trastuzumab were diarrhea, anemia, and neutropenia. In the APHINITY trial, the rate of grade 3 or higher diarrhea was 9.8% in patients receiving pertuzumab compared to 3.7% in the placebo group. However, rates of high-grade diarrhea of up to 37% were reported in a retrospective study of patients receiving pertuzumab in the neoadjuvant or metastatic settings [23]. Congestive heart failure attributable to pertuzumab is rare [24]; however, it was more common in those receiving pertuzumab (0.6%) versus placebo (0.2%) in APHINITY [9].

While the improvement in iDFS with adjuvant pertuzumab demonstrated in the APHINITY trial may be considered modest, its use was predicted to be cost-effective based on quality-adjusted life years gained by preventing disease recurrence [25]. However, the addition of potential treatment-related toxicity is likely to impact treatment decisions as clinicians weigh the individualized benefit in reduction of risk of recurrence with toxicity. Further work to identify factors beyond node-positive disease that may predict a high risk of metastatic recurrence following chemotherapy and trastuzumab and increased benefit from adjuvant pertuzumab are likely planned as the APHINITY trial data continue to mature and will be helpful in making these decisions in the future.

In contrast to pertuzumab, the majority of patients in our dataset did not go on to receive adjuvant neratinib, although its use was more common in node-positive patients. Our analysis may be limited by short-term follow-up of patients for at least 3 months following completion of adjuvant trastuzumab to determine if adjuvant neratinib would be used. The ExteNET trial allowed patients to enroll within 12 months of completing adjuvant trastuzumab; however, the benefit was greater in patients that initiated therapy closer to completion of adjuvant trastuzumab, particularly within 6 months [10, 26, 27].

In the ExteNET trial, diarrhea was the most common neratinib-related adverse event with > 40% of patients experiencing grade 3 or 4 diarrhea. Patients were offered loperamide as needed for symptomatic management, though prophylaxis was not initially administered per protocol [10]. Neratinib-related diarrhea typically occurs within the first cycle of therapy and often leads to dose reductions in the absence of anti-diarrheal prophylaxis [28]. We now know based on the results of the CONTROL trial that scheduled loperamide plus colestipol is effective anti-diarrheal prophylaxis in patients receiving neratinib [29]. In our study, all patients receiving neratinib received loperamide, and about two-thirds also received prophylactic colestipol. This is likely a result of provider education and standardized therapy care plans that include anti-diarrheal prophylaxis that are used across our practice sites. In further contrast to the utilization of adjuvant pertuzumab, Schwartz et al. found that adjuvant neratinib was not cost-effective, even in this high-risk group, based on the 5-year data from the ExteNET study [30]. Patients and providers may have concerns about potential toxicity with neratinib and a modest decrease in the risk of recurrence and ultimately choose not to initiate therapy.

Very few patients received both adjuvant pertuzumab and neratinib in our study which is consistent with a lack of data for the benefit of adjuvant neratinib in patients receiving adjuvant pertuzumab and vice versa.

Our study has several limitations including a relatively small sample size and treatment within one hospital system with standardized cancer therapy administration order sets. Patients were followed for only 3 months after completion of adjuvant trastuzumab; therefore, the rate of adjuvant neratinib administration may be underestimated. Ongoing follow-up of this cohort of patients is planned to further understand the shifting practice patterns of adjuvant therapy in HER2-positive breast cancer with the availability of new agents and potentially validation of patient selection strategies to maximize benefit and minimize toxicity for patients less likely to experience recurrence.

## Data Availability

The datasets generated during and/or analyzed during the current study are available from the corresponding author on reasonable request.
